# Racially fair pupillometry measurements for RGB smartphone cameras using the far red spectrum

**DOI:** 10.1038/s41598-023-40796-0

**Published:** 2023-08-24

**Authors:** Colin Barry, Edward Wang

**Affiliations:** 1 Electrical and Computer Engineering Department, University of California San Diego, La Jolla, CA USA; 2 Design Lab, University of California San Diego, La Jolla, CA USA

**Keywords:** Biomedical engineering, Biomarkers

## Abstract

Pupillometry is a measurement of pupil dilation commonly performed as part of neurological assessments. Prior work have demonstrated the potential for pupillometry in screening or diagnosing a number of neurological disorders including Alzheimer’s Disease, Schizophrenia, and Traumatic Brain Injury. Unfortunately, the expense and inaccessibility of specialized pupilometers that image in the near infrared spectrum limit the measurement to high resource clinics or institutions. Ideally, this measurement could be available via ubiquitous devices like smartphones or tablets with integrated visible spectrum imaging systems. In the visible spectrum of RGB cameras, the melanin in the iris absorbs light such that it is difficult to distinguish the pupil aperature that appears black. In this paper, we propose a novel pupillometry technique to enable smartphone RGB cameras to effectively differentiate the pupil from the iris. The proposed system utilizes a 630 nm long-pass filter to image in the far red (630–700 nm) spectrum, where the melanin in the iris reflects light to appear brighter in constrast to the dark pupil. Using a convolutional neural network, the proposed system measures pupil diameter as it dynamically changes in a frame by frame video. Comparing across 4 different smartphone models, the pupil-iris contrast of N = 12 participants increases by an average of 451% with the proposed system. In a validation study of N = 11 participants comparing the relative pupil change in the proposed system to a Neuroptics PLR-3000 Pupillometer during a pupillary light response test, the prototype system acheived a mean absolute error of 2.4%.

## Introduction

In this paper, we introduce a novel method of capturing pupillometry data on smartphone RGB cameras. Pupillometry is a measurement of pupil diameter, often performed as a dynamic measurement in response to a stimulus, such as light, sound, critical thinking tasks, or emotion-inducing images. The pupil’s response to the stimuli provides insight into neurological functions^1,2^. As such, pupillometry measurements are often included in neurological tests, traumatic brain injury screening, and intracranial hypertension monitoring^3–5^. Additionally, recent research has demonstrated significant correlations between pupil responses and a number of neurological diseases including Traumatic Brain Injury, Alzheimer’s Disease, Parkinson’s Disease, opioid use, Schizophrenia, and Mild Cognitive Impairment^6–13^. Thus, pupillometry provides a valuable quantitative biomarker for neurological conditions that can be difficult to measure directly.

This work hopes to facilitate the use of pupillometry and related research areas through vastly expanding access to pupil dilation measurements. Currently, the multi-thousand dollar cost of pupillometers restricts the use of pupillometry outside of high resource institutions even though, pupillometry has significant potential beyond clinical laboratories. Prior work demonstrates that pupillometry has potential for prognostic assessments from acute head injuries, at-home cognitive screening in older adults, or opioid testing^4,7,13–15^. However, the lack of pupillometry resources contributes to a lack of research on at-home pupillometry tests, test retest reliability studies of novel pupil biomarkers, and high temporal resolution studies of multi-day or multi-week studies. To expand pupillometry access, we propose a novel method of enabling everyday smartphone RGB cameras to perform pupillometry measurements.

The key to accurate pupillometry is capturing images in which the pupil can be clearly differentiated from the iris. However, segmenting the pupil and the iris is difficult in visible light, especially for individuals with darker irises^1,14^. The pupil acts like an aperture allowing light to pass through to the back of the eye, where most of the light is absorbed, thus the pupil appears black. Clinical pupillometers and other high performance pupillometry systems utilize near-infrared light to measure the pupil because melanin of the iris reflects longer wavelengths and appears bright, which significantly contrasts the dark pupil. Near infrared imaging is not possible in smartphone RGB cameras because smartphone cameras have built-in filters that cutoff light outside the visible spectrum to improve image quality for photography. This paper addresses the challenge of performing pupillometry within the light spectrum of commodity RGB cameras. We focus on smartphone cameras as the most ubiquitous cameras for individuals and consumers.

Ubiquity, or the ability of our solution to generalize across smartphones, is a key goal of the paper. There are a number of inherent difficulties to proliferating a solution across smartphone devices with different components, interfaces, and physical designs^16^. The vision is to expand pupillometry access to anyone from high school sports coaches checking students for concussions to neurological screening tasks for older adults. Leveraging smartphones enables the measurement to be inexpensive by relying on existing smartphone hardware and software; however, this approach is only realistic if it can function on nearly every smartphone, including cheap smartphones.

**Figure 1:**
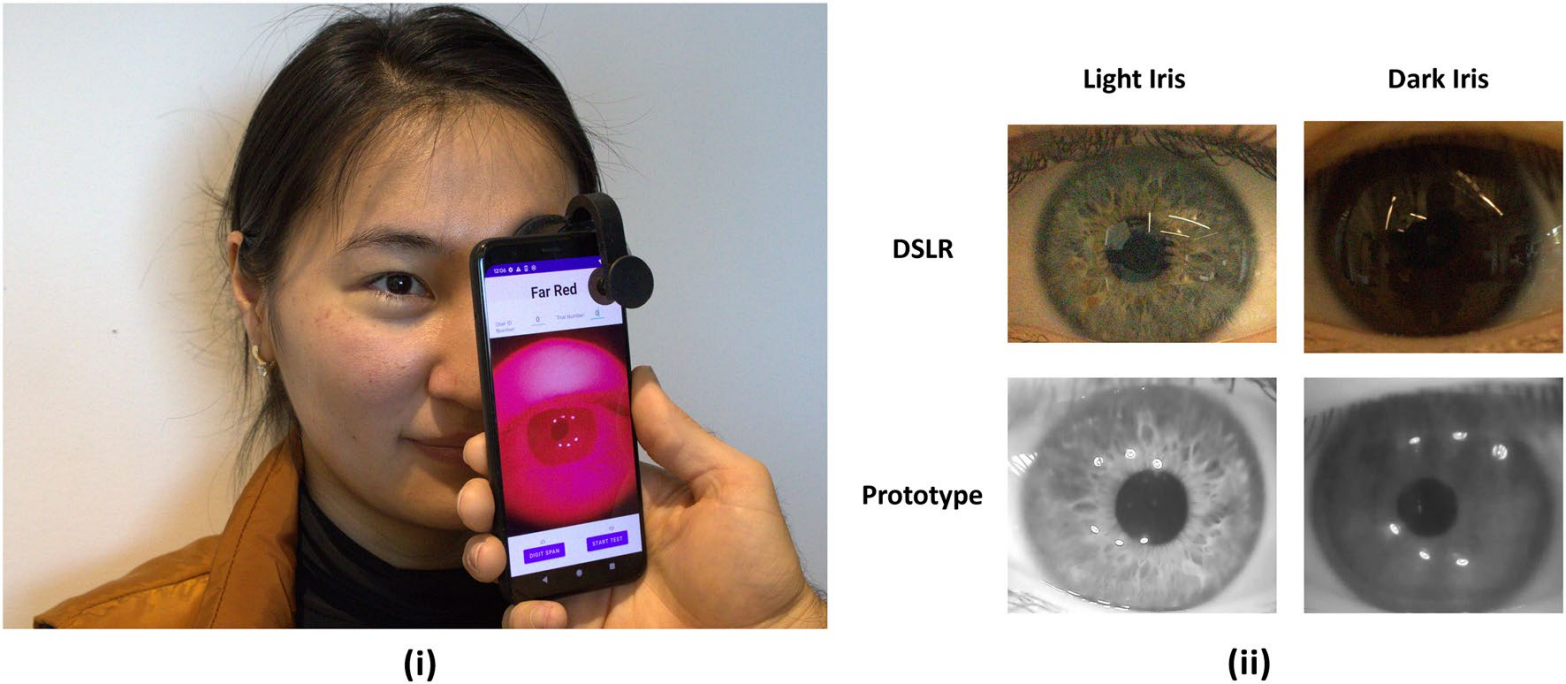
Overview of Pupillometry System for Commodity Smartphone Cameras: Part (i) exemplifies the use of the prototype system on a commodity camera. Part (ii) Light iris and dark iris images from a a full spectrum DSLR camera and the target spectrum prototype system,, where the images from the prototype spectrum significantly improve the ability to distinguish the pupil in the dark iris image.

Although prior research on smartphone pupillometry exists, we have been unable to find any prior work that functions across smartphone models and fundamentally improves the pupil to iris contrast in RGB camera images. Most prior work in smartphone-based pupillometry simply utilize convolution neural network (CNN) models to attempt to segment the pupil from the iris, but these works are fundamentally limited by the pupil to iris contrast of the smartphone cameras images^3,17,18^. These approaches work very well for individuals with blue or light colored irises, but, even in controlled lighting conditions with CNN models, it is extremely difficult to segment the pupil from a dark brown iris^3,18^. As such, most prior works on smartphone-based pupillometry either exclude individuals with dark iris colors from the analysis or report poor performance because the iris and the pupil are difficult to distinguish in the full visible spectrum^3,17,18^.

One prior work successfully demonstrates accurate pupillometry utilizing the Near Infrared (NIR) Facial recognition cameras included in some smartphones^14^. Unfortunately, this NIR camera was previously only available in expensive phone lines and it is now phased out of most major smartphone brands including Google and Motorolla. Also, brands that previously or currently do include the facial recognition camera, generally do not expose the camera to the developer so the proposed use of NIR facial recognition cameras is not feasible. As such, no prior works meet the challenges performing accurate pupillometry and generalizing well across the variety of existing smartphone devices^16^. This paper presents a novel methodology to perform smartphone pupillometry that significantly increases the pupil to iris contrast and only requires a smartphone to include an RGB camera, which is included in the smartphones of every major brand.

In the preliminary studies, a custom prototype suggests the novel methodology could vastly expanding access to pupillometry. The vision is to expand pupillometry based testing for concussions, drugs, and other neurological insights to homes, schools, or sporting events. Our proposed novel pupillometry method utilizes filtered far red wavelengths within the visible spectrum to significantly differentiate the pupil and the iris in RGB cameras. Figure 1 demonstrates the effect of the proposed pupillometry method. For the scope of this paper, we define the far red spectrum to be the longer wavelengths of red light within the visible spectrum at approximately 630–700 nm. Utilizing the far red light spectrum extends some benefits of infrared, clinical pupillometers to commercial cameras and smartphones.

This novel pupillometry method is implemented in a prototype smartphone attachment containing LEDs and a longpass filter to ensure the images contain only the target spectrum of approximately 630–700 nm. The prototype leverages the smartphone camera, flashlight LED, and a custom application to perform a pupillary light reflex test. With the prototype attached, the smartphone records the participant’s pupil response as the smartphone flashlight provides a bright light stimulus. The prototype with its components is depicted in Fig. 3. The functional prototype demonstrates that this filtered spectrum is suitable for pupillometry and achievable on nearly all commodity smartphone RGB cameras.

In validating the accuracy of the novel measurement, pupillary light reflex tests are performed. Pupillary light reflex (PLR) tests involve stimulating the pupil with bright light to envoke a large pupillary constriction. The PLR test is chosen because it provides a common and valuable biomarker with significant potential for neurological screening, especially for TBI. The PLR light stimulus is provided by the smartphone-based prototype by using an application that controls the smartphone flashlight. The prototype contains a neutral density filter to lessen the intensity of the flashlight. Pupil measurement of N = 12 participants with the prototype is recorded simultaneously with a clinical pupillometer as a ground truth measurement. The validation results in an MAE of 2.4%, which improves upon the current state of the art for smartphone pupillometry.

The generalizability of the pupillometry method is assessed across 4 smartphone models of different manufacturers. We demonstrate that the prototype plastic attachment with LEDs and light filters can be attached to all tested smartphones to enable accurate pupillometry measurements. On each smartphone model, video frames of (N = 11) pupils taken in the far red lighting condition are compared to a normal lighting condition. The pupil to iris contrast yields an average 451% increase across the smartphones with the far red lighting.

## Results

### Concept

**Figure 2:**
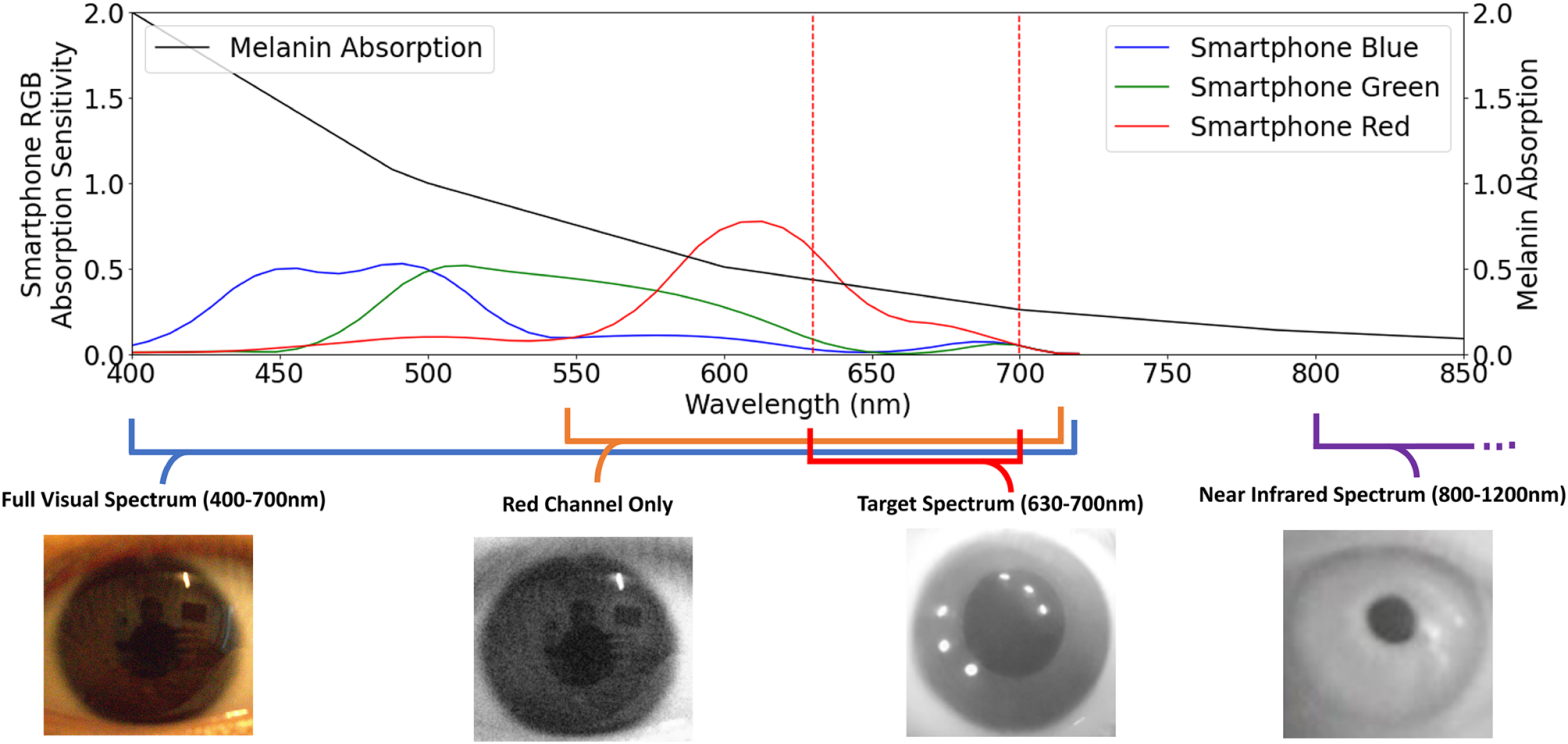
Four images of the same dark iris eye in different light spectrums is shown to allow the user to see the difference in pupil to iris contrast in different imaging conditions. The imaging spectrums include the full visual spectrum (400–700 nm) and red channel (approx. 550–700 nm), both from a DSLR RGB camera. The target spectrum proposed in this paper (630–700 nm), identified with dotted red lines, is displayed as captured from the prototype system. The ideal near-infrared spectrum for pupillometry (approx. 800–1200) is captured from the system described by Barry et al^14^. The melanin absorption at different wavelengths as found by Keilhauer et al^19^ is plotted in black with the target spectrum indicated with red dotted lines. Additionally, the absorption of the red, green, and blue channels of an smartphone camera characterized by Ji et al^20^ to exemplify the absorption of the red and full visible spectrum in smartphone cameras.

In this work, we propose using the far red edge of the visible range to perform pupillometry. The main purpose of the prototype attachment is to enable commodity smartphones to image exclusively in the far red wavelength spectrum (approximately 630–700 nm), where the pupil and iris absorption differs. This spectrum is at the edge of the visible range, and subsequently within the passband of most commodity RGB camera filters. Importantly, the melanin absorption in this far red spectrum is significantly less than the rest of the visible spectrum so the iris appears much lighter, even for individuals with high melanin and dark brown irises. Without augmentation, the red channel of the smartphone is broadband, such that shorter wavelength light absorbed can cause low pupil-to-iris contrast. The longpass filter in the proposed attachment allows the smartphone to image specifically within the wavelengths of interest to differentiate the pupil from the iris, as shown in Fig. 2.

### Prototype

The prototype is designed as a universal attachment to fit most modern smartphones with the assumption that the back camera is within 2.5 cm from the edge of the phone. The attachment can be attached and adjusted to fit smartphones with any variation of positioning between the camera and flash LED. The prototype is a self contained attachment housed within a 3D printed circular casing that attaches to any smartphone via a 3D printed fastening screw. The 3D printed attachments contains 4 critical components: a 630 nm longpass filter (Edmund Optics SCHOTT RG-630, 12.5mm Diameter 3mm Thick Colored Glass Longpass Filter), a custom circular PCB board with 625 nm LEDs (Marubeni 625SMT LEDs), a 15x macro lens, and a 3D printed rubber eye cup. To enable imaging within the target spectrum of approximately 630–700 nm, the 630 nm longpass filter blocks out light shorter than 630 nm. The longpass filter sits at the back of the attachment, such that it sits just in front of the smartphone camera when properly attached. Wavelengths above approximately 700 nm are increasingly attenuated by the filter included in the smartphone camera. Thus, we convert a common smartphone camera into a pupillometer imaging in the 630 to approximately 700 nm range. Given that pupillometry involves imaging the eye at close range, a macro lens fits in the attachment on top of the filter to adjust the focal length and magnify the pupil for accurate size estimation.

**Figure 3:**
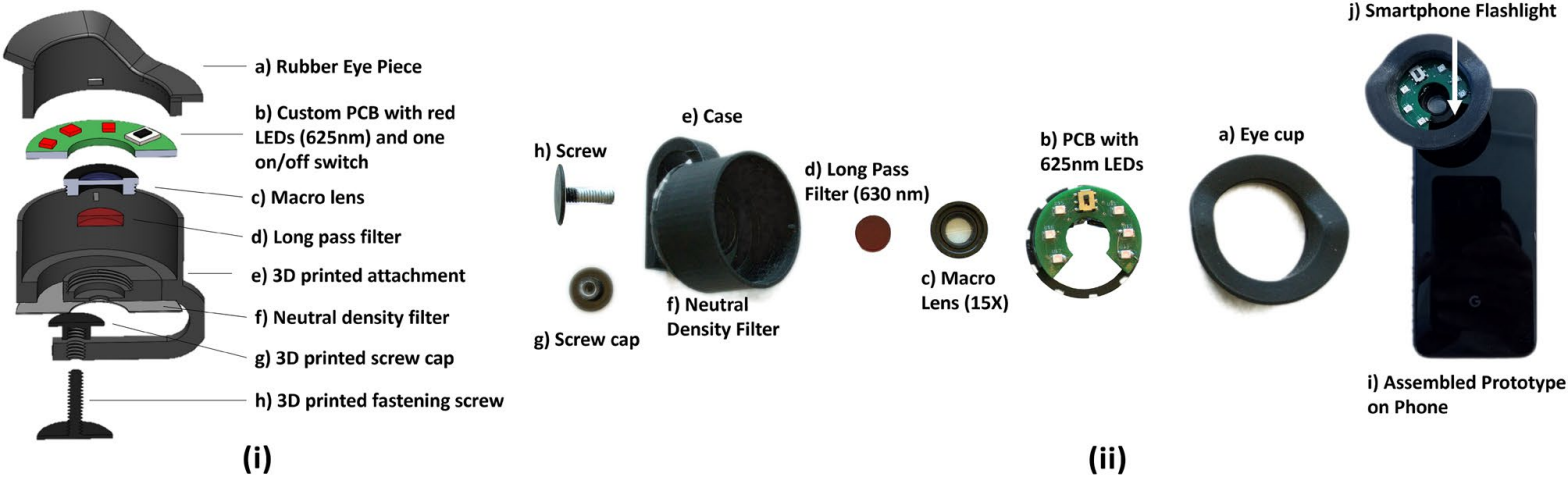
Components of Prototype Smartphone Attachment: (**a**) Rubber eye cup; (**b**) PCB with 625 nm LEDs, on/off switch, coin cell battery (on backside), and 3D printed mounting structure; (**c**) Macro lens; (**d**) 630 nm long pass filter; (**e**) 3D printed case to house all components; (**f**) Neutral density filter on the back of the circular case to prevent excessive light exposure from the smartphone flashlight; (**g**) 3D printed screw cap; (**h**) 3D printed screw for fastening attachment to smartphone; (**i**) Fully assembled prototype on the smartphone; (**j**) Smartphone flashlight LED used for light stimulus in pupillary light response test.

The design of the prototype involves no assumptions about a user’s lighting environment. The prototype creates a proper lighting environment by illuminating the field of view with 6 Marubeni SMT 625 nm LEDs, all with a spectral half width of 25 nm. The LEDs are controlled by a simple switch and powered by a button cell battery on the back of the PCB embedded in the attachment. Note that any illuminating light outside of the target 630–700 nm range is attenuated by longpass and smartphone camera filters. The PCB is shaped as a semi-circle such that it swiveled to position the gap over the smartphone flashlight, assuming the the flashlight is within approximately 1.5 cm of the camera. The flashlight is meant to serve as a stimulus for the user to invoke pupil constriction. The flashlight is controlled by the smartphone application to follow procedures of common pupillary response tests. Additionally, the back of the 3D printed case includes a neutral density filter to cover the smartphone flashlight such that the LED can be safely shined at the participant’s eye without excessive light exposure. The rubber eye cup blocks outside light to ensure light from the environment does not contribute unexpected stimuli or glare in the readings. Also, the scope serves as a usability aid in positioning the camera correctly in 3D space to appropriately focus on and measure the pupil.

### Validation

**Figure 4:**
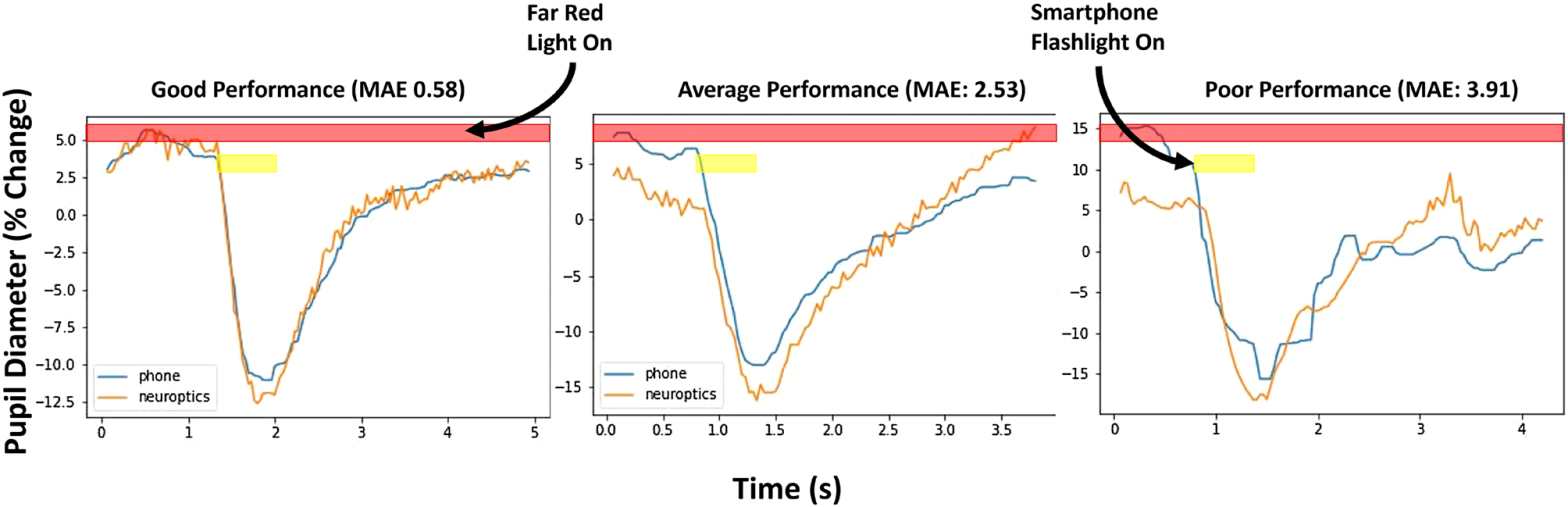
This figure offers 3 plots for comparing pupillary light reflex responses recorded with the proposed smartphone system and Neuroptics pupillometer. The plots are labelled for good, average, and poor performances representative of the system performance. Also, red and yellow shading indicate the times when the when far red light and full spectrum smartphone flashlight respectively illuminate the eye.

**Figure 5:**
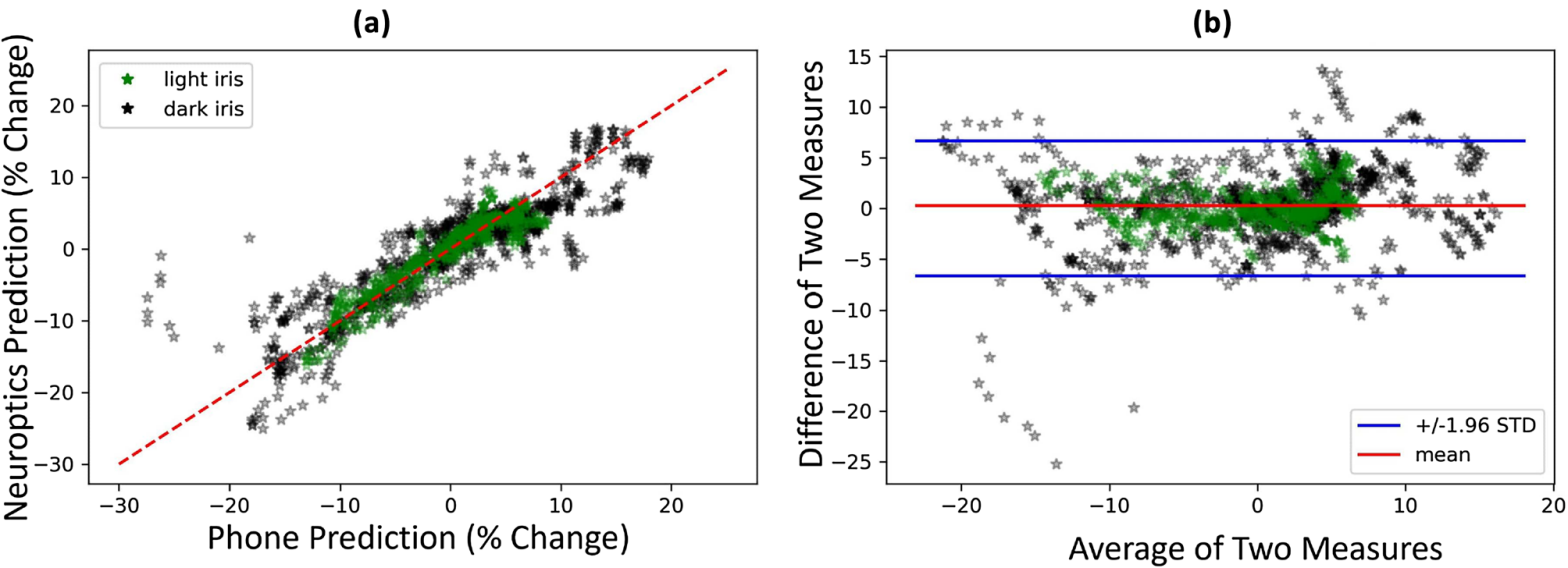
(**a**) Regression plot of N = 12 participants and (**b**) Bland Altman plot of N = 12 participants.

The smartphone-based pupillometer is compared to a Neuroptics PLR 3000, a clinical gold standard for pupil measurements. The PLR test records the pupil response to a 1s flash of light. The test showcases the performance of the smartphone system during a large, fast change in pupil size. Figure 4 exemplifies typical performances from the prototype system compared to the Neuroptics pupillometer. Figure 5 provides the regression and bland altman plots to demonstrate the performance of the system on N = 12 participants with an average pearson’s correlation of 0.93, a mean absolute error of 2.25%, and a standard deviation of 1.3%. Note that one participant (P12) is excluded from this analysis due to errors in the Neuroptics pupillometer readings.

## Cross device compatibility

We compare the performance of the novel smartphone pupillometry method on smartphone devices of different manufacturers to ensure functionality across a range of devices with different operating systems, hardware configurations, cameras, and physical form factors. The pupil-to-iris contrast is used to determine performance on the Google Pixel 4, Apple iPhone 12, Samsung Galaxy A53, and Motorolla Moto G Power 2022. For each phone, the pupil to iris contrast is calculated for video frames illuminated with the full visual spectrum and for pupil video frames illuminated with the target far red spectrum. Note that two participant (P11 and P13) did not participate in the cross device compatibility study. Table 1 reports the pupil to iris contrast values for all 11 participants. The average percent increase in contrast across all participants and all smartphones is 451%. The average increase in contrast for dark brown eye participants only is 624%.

**Table 1:** Cross Phone Comparions: The performance across smartphone devices is compared by calculating percent change in pupil iris contrast between video frames in full visual spectrum and video frames in the target far red spectrum.

ID	Pupil Iris contrast percent increase						Eye color		
	A53	Iphone12	Moto G	Pixel4	Mean	STD	DSLR contrast	Iris Luminance	Description
1	111.3	106.6	26.7	426.8	186.7	211.8	89	116	Blue
2	252.9	215.1	202.6	161.2	193.0	28.2	47	104	Blue
3	80.9	6.1	125.7	142.3	91.4	74.3	60	84	Blue
4	137.3	175.7	225.0	−29.9	123.6	135.2	31	63	Light brown
5	500.3	962.6	1956.2	205.4	1041.4	878.0	26	55	Dark brown
6	159.2	154.5	381.2	131.1	222.2	138.2	22	45	Dark brown
7	231.1	103.3	−65.7	277.2	104.9	171.5	20	42	Dark brown
8	185.7	941.9	597.2	107.7	549.0	419.2	13	36	Dark brown
9	180.6	310.9	482.6	349.1	380.9	90.1	15	35	Dark brown
10	216.7	396.1	3464.6	225.9	1362.2	1822.7	14	34	Dark brown
12	1579.2	134.8	2232.8	−232.9	711.6	1330.2	9	26	Dark brown

## Discussion

The pupillometry measurements here could be greatly expanded in future works. This work focuses on the application of PLR tasks because light response tests are commonly used in both clinical and research settings. The PLR tasks demonstrate that far red light does not impede the ability of measuring the pupil response to white light stimulus. Other common tasks including digit span recall tasks, oddball auditory tasks, or emotion-based tasks are feasible with a smartphone-based system. A key potential benefit of a smartphone-based system is the high plasticity of smartphone applications that allows for continued ingenuity in providing new and different stimuli. The smartphone can be programmed to provide a wide variety of visual or auditory stimuli for a pupil response task.

As a smartphone-based system, usability and comprehension features can be added within an application. It is possible to imagine different use cases of smartphone pupillometry with different usability features. For example, the current design assumes assisted measurements with one user performing a measurement on a second individual; however, modifications could be made to outfit the scope on to the front facing RGB camera to facilitate self administered measurements. Prior works in smartphone pupillometry have demonstrated the usability of smartphone pupillometry in older adult users, suggesting possibilities for this type of technology within at-home deployments^14^.

A key limitation of the smartphone device is that it is not capable of measuring absolute pupil measurements. Absolute pupil size, often recorded in millimeters, could be utilized as a feature; however, absolute pupil size is highly dependent on light exposure and physiological conditions. As such, absolute pupil size measurements may have poor repeatability so pupillometry studies often use relative size or a metric of change from a baseline reading, especially for measuring the pupil response to a stimulus^7^. The proposed prototype can only measure percent change or other relative measurements because accurate absolute size measurements requires a distance measurement between the camera and the pupil. Some prior works have reported absolute size by assuming all subjects have the same facial structure with respect to eye depth; however, this is a poor assumption that has not been validated.

The prototype is also limited by the inability to account for eye movement. Currently, the pupil measurements do not correct for eye movement and are prone to errors when participants look away from the camera. This limits the potential for certain types of pupil response test that may require spatial stimulus. Future plans for the prototype include resolving this issue by including eye tracking features and accounting for eye movement during pupil measurement tasks. This would not only improve the pupil measurement accuracy, but also enable an entirely new measurement dimension of eye tracking studies within the same prototype. Note that eye tracking does not necessarily require accurate pupil segmentation and prior work on eye tracking on smartphones exists^21,22^.

Future exploration of smartphone pupillometry utilizing the proposed methodology may consider standards or calibrations to improve the measurement consistency across smartphone devices. Although the proposed methodology demonstrates the potential for nearly all smartphones to accurately perform pupillary light response measurements with an RGB camera, differences in components may cause unequal measurements. For example, if the smartphone flashlight LED component differs significantly between smartphone models, the difference in luminance and light spectrum during the light stimulus may cause variations in the response. Also, the light history from the environment of a participant may affect the pupil response, such that measurements performed during a sunny day and a dark night may be different for the same individual^23,24^. Exploring diagnostic potential and ensuring measurement consistency across environments and smartphone models requires further studies and possibly further innovation.

## Methods

### Smartphone application

A custom smartphone application is developed to administer pupil response test stimuli while simultaneously recording the pupil response. The goal of the application is to enable a user to perform a pupil response tests on other individuals. This application, shown in Fig. 1 includes a camera preview of the eye to position the smartphone correctly before starting the tests. The user is meant to position the pupil clearly within the camera frame with the scope touching the test participant’s face. To facilitate single hand use of the smartphone for test administration, the physical volume buttons on the side of the smartphone can be used to start a pupil response test. During a pupil response test, the application displays real time video with a testing in progress label on the on-screen buttons.

### Participant eye color

Participants were recruited to have a bias towards darker eye colors because darker eye colors are more difficult to differentiate from the pupil in the visible spectrum based on prior work^3,18^. The participant eye color is defined qualitatively by the participant’s self assessment of their eye color in the following categories: light/blue, hazel/light brown, and dark brown. The eye color is also quantitatively characterized by the iris luminance based on an image from a digital single reflex lens (DSLR) camera (Camera: Cannon EOS R6 Mark II, Lens: Cannon RF 35mm). The DSLR camera provides a single, high quality image for analysis. Within an image, a 15x15 pixel area of the iris is manually selected for analysis. The median luminance of the selected iris pixels are reported for iris. Luminance is calculated by converting the RGB image to the CIELAB color space to quantify iris color as similarly performed in prior work^25^. The participant demographics and eye colors are available in Table 2. Participants 11 and 13 did not participate in the cross device compatibility study. Also, participant 12 was excluded from the validation study because of erroneous measurements in the ground truth neuroptics device.

Note that smartphone videos and images were specifically avoided for this measurement. The computation photography integrated in smartphones can significantly alter image color by increasing local and/or global dynamic range. The DSLR camera is preferable over smartphone videos for the image quality.

**Table 2:** Participant demographics.

Participant ID	Gender	Age	Ethnicity	Eye color description	Iris luminance
1	M	26	White	Blue	116
2	M	25	White	Blue	104
3	M	26	White	Blue	84
4	F	28	Hispanic/Latino	Light brown	63
5	M	24	Asian	Dark brown	55
6	M	26	Asian	Dark brown	45
7	M	25	Asian	Dark brown	42
8	M	28	Asian	Dark brown	36
9	M	21	Asian	Dark brown	35
10	M	29	Asian	Dark brown	34
11	F	30	Asian	Dark brown	31
12	F	23	Asian	Dark brown	26
13	M	23	Asian	Dark brown	18

### Validation experiment

In the validation experiment, the smartphone-based system is compared to the Neuroptics PLR 3000. For the study comparison, the smartphone system records from the left eye, while the Neuroptics device simultaneously records from the right eye. The Neuroptics device records absolute pupil size in millimeters at 30Hz while the prototype smartphone system records pupil size in pixels at 60Hz. The measurements are aligned in time by downsampling the smartphone data points to 30Hz and excluding blinks from both devices. The two measurements are then compared using percent change from mean. The Neuroptics device does not have the capacity to be controlled remotely so the recordings are started by simultaneous button press. As such, the recordings starts can be misaligned so some recordings are manually aligned in post-processing. Both devices record for exactly 5 second. While the smartphone administers a 5 second PLR test, the Neuroptics device records, but provides no stimulus. The smartphone provides the only stimulus during the test.

The PLR test involves a 5 second recording with a short light stimulus. For this paper, the PLR test protocol is as follows: 1 second of no light, 1 second of light stimulus provided by the camera flashlight, 3 seconds of no light. Note that the smartphone flashlight LED is covered by the neutral density filter of the prototype to avoid excessive brightness in the light stimulus. During the entirety of the PLR test, the far red LEDs of the prototype illuminate the eye for imaging. No other sound or stimuli are included in the study.

This test is chosen due to its application and versatility in pupillometry. Researchers and clinicians commonly use this test to investigate a number of neurological conditions including traumatic brain injury, cognitive impairment, and substance use. The PLR test within the validation experiment demonstrates functionality across a range of pupil sizes and provides a comparable measurement to prior work^3,14,18^. The experiment and subject recruitment presented here are approved by the UC San Diego Internal Review Board (IRB #200201).

### Far red light stimulus

The system is designed to constantly illuminate the eye with 625 nm light. Providing this far red light before the white light stimulus allows the participant’s pupil to be adapted to the far red light such that the measurement records only the response to the white light. Unlike white light or blue light, red light does not provide a strong stimulus for pupil constriction. Prior work demonstrates that red light alone causes far less pupillary constriction compared to a combined blue and red light condition^26^. The work suggests that, although red light will cause some constriction of the pupil, pupil response tasks will not be significantly limited. The red light is a sufficiently weak stimulus for the pupil, such that the pupil maintains significant capacity to constrict in response to other stimuli. Also, the lighting is constant for the duration of the testing so it does not evoke changes within the pupil during the test. Therefore, pupil response test involving other visual, cognitive, or emotional stimuli can be performed without obstruction.

### Cross device validation

The cross device validation compares performance of the prototype system across 4 smartphone models representative of a variety of manufacturers, operating systems, hardware configurations, and prices. The smartphone models are as follows: Google Pixel 4, Apple iPhone 12, Motorolla Moto G Power 2022, Samsung Galaxy A53. The validation utilizes the pupil to iris contrast in recorded video frames as the key metric for comparison. This metric represents the ability to discern the pupil from the iris. The measurement is performed on all four phones for each participant. For each phone, the pupil to iris contrast in calculated in two conditions meant to compare the proposed prototype of this paper with the normal visual spectrum. Condition 1 includes the standard prototype described in 2.2 with far red LEDs and a long pass filter. Condition 2 includes the prototype WITHOUT the far red LEDs or the long pass filter. For condition 2, the pupil is illuminated only with the smartphone camera covered by the neutral density filter of the prototype. Both conditions include the macro lens and the eye cup attachment.

1 $$\begin{aligned} Contrast \% Increase = \frac{PupilIrisContrast_{far red} -PupilIrisContrast_{full}}{|PupilIrisContrast_{full}|} \end{aligned}$$

In computing the Pupil Iris Contrast Percent Increase in Table 1, we use Eq. (1), where

$$\begin{aligned} Pupil Iris Contrast = Iris Pixel Intensity - Pupil Pixel Intensity \end{aligned}$$.

#### Pupil Iris contrast calculation

The pupil iris contrast of an image is determined by comparing the light intensity of the pupil to the light intensity of the iris. This calculation is performed on video frames from the each smartphone with and without the full prototype system. Video frames are converted from RGB to grayscale by selecting the red channel of the image only. The red channel contains the wavelengths of interest so it holds the majority of the signal. From the red channel, 15 by 15 blocks of 225 representative pixels are selected from the pupil and the iris for color contrast comparison. The pixels are chosen as the center pixels of the pupil. From the center of the pupil, the iris pixels are chosen from approximately 2 times the radius at an angle of 45 degrees. Adjustments to the selected pixel locations are made to avoid glare that may interfere with the calculations. The difference in pixel intensity between the pupil and iris is reported as the contrast.

### Data processing

The prototype system records videos of the eye during a pupillometry test then uses a convolutional neural network for segmenting the pupil and iris. After the smartphone records the pupillometer video, the data is offloaded onto a secure server for processing. The recorded video is converted to frame by frame pupil diameter values. Each frame of a video is down sampled and converted to a single channel grayscale image used as an input to the PupilLocator network proposed by Eivazi et al^27^. with minor modifications. The CNN outputs pupil diameter and position values. Videos pupil predictions overlayed on original recorded videos are available in the supplemental material.

For the Neuroptics PLR-3000 system, the pupil diameter values are directly provided along with blink detection estimates. The Neuroptics device does not provide insight or alterations for the pupil diameter estimation methodology. From the Neuroptics data, the data points that are detected as blinks are excluded in the analysis. To avoid characterizing possible errors from the prototype system as blinks, the data points from the prototype system corresponding to the detected blinks in the Neuroptics system are excluded.

## Conclusion

Commodity RGB cameras on smartphones provide an ideal platform for expanding the use and research of pupillometry outside of high resource clinics. In this paper, we propose a solution to enable pupillometry measurements on RGB smartphone cameras by leveraging a novel methodology of imaging the pupil in the far red light spectrum. This methodology is validated with a prototype attachment to demonstrate the success of the new methodology, especially on individuals with dark irises. We expect that this prototype could be improved and simplified for low cost production.
